# Pausing TPN to Decrease Abnormal Newborn Screens: A NICU Quality Initiative

**DOI:** 10.1097/pq9.0000000000000595

**Published:** 2022-09-15

**Authors:** Jaclyn B. Wiggins, Marium Khan, Brooke D. Vergales

**Affiliations:** From the * Division of Neonatology, University of Virginia, Charlottesville, Va.; † Department of Pediatrics, University of Virginia, Charlottesville, Va.

## Abstract

Detection of metabolic and genetic disorders via the newborn screen (NBS) remains a great diagnostic achievement in medicine. Unfortunately, many false positives for neonates due to acute illness lead to repeat testing. For example, the rate of abnormal NBS in very low birth weight (VLBW) neonates at the University of Virginia was 66%, with 23% due to total parenteral nutrition (TPN) on the amino acid profile.

**Methods:**

This study describes a quality improvement (QI) initiative completed in the NICU at a quaternary care center. The primary and secondary outcomes were the percentage of abnormal NBSs in VLBWs and all admissions. The intervention required a pause in TPN, and a dextrose-containing fluid ran for 4 hours before collecting the NBS. During PDSA cycle 1, the TPN pause occurred at 1400, and the collection of the NBS occurred at 1800. During PDSA cycle 2, we aimed to decrease the number of blood draws per neonate and, thus, paused the TPN at 0000 to enable NBS collection at 0400 with routine morning laboratory work.

**Results:**

The rate of abnormal screens in VLBWs decreased from 66% to 49%; *P* < 0.006; 95% CI, 0.04–0.27, and the rate of abnormal screens in all admissions dropped from 45.2% to 28.8%; *P* < 0.0001; 95% CI, 0.06–0.51. Hospital costs decreased from $244.79 to $170.86 per patient in the cost of the NBS cards alone.

**Conclusion:**

By pausing TPN for 4 hours before drawing the NBS, we decreased the number of abnormal NBS in all admissions while also decreasing hospital costs.

## INTRODUCTION

The newborn screen (NBS) is a required test for all newborns. Initially developed in the 1960s, the NBS to screen for phenylketonuria has expanded over the past 50 years and screens for over 30 metabolic and hereditary conditions in Virginia between 24 and 48 hours after birth.^1^ In Virginia, a repeat NBS is not routinely sent at more than 8 days. Therefore, sending the test early to diagnose and treat conditions promptly is important. Many conditions, such as phenylketonuria, require special formulas to prevent neurodevelopmental impairment. Earlier diagnosis allows for timely, appropriate nutrition and less neurologic impairment. In Virginia, roughly 6 per 1000 screening tests are positive and lead to a diagnosis.^1^ However, as it is a screening test, approximately 1 in 300 infants will have a false-positive result.

In premature neonates, the false positive rate is even higher due to their unique metabolic needs.^2^ In the first week after birth, very low birth weight neonates (VLBW, <1500 g birth weight) need high protein supplementation via total parenteral nutrition (TPN) to prevent catabolism.^2^ This protein supplementation leads to higher-than-normal amino acid levels in the blood; thus, an NBS drawn 24 hours after birth can have a falsely elevated amino acid profile. If the first screen is abnormal, then a follow-up screen or plasma amino acids and urine organic acids are sent to determine whether the screen was a true or false positive.^3^ These tests take significant time to result and cause the patient’s family to have unnecessary stress. In addition, each Virginia NBS blood spot card costs the hospital $138.00.^1^ Our unit identified that 94% of our VLBW population are on TPN at the time of their NBS, and therefore, they are at risk for having a falsely elevated amino acid profile.

Other hospitals have published that pausing TPN before obtaining the NBS has decreased the false positive rate for those screens with an abnormal amino acid profile.^4–6^ At Children’s Hospital of Orange County, by pausing TPN 3 hours before drawing the NBS, Morris et al^4^ reduced the abnormal NBS rate by 74%, leading to a significant decrease in blood draws for neonates and cost savings for the hospital. They estimated that the new protocol saved more than 80% of healthcare costs due to a decrease in NBS cards. Another study reduced the false-positive NBS rate by pausing the TPN and running a dextrose-containing fluid for 4 hours before drawing the NBS. Abnormal results decreased from 66.7% to 35% in VLBW infants and 20.6% to 10.7% for all NICU admissions.^6^

Therefore, a multidisciplinary team at University of Virginia (UVa) created a new standard workflow to pause TPN before obtaining the NBS to decrease the rate of falsely elevated amino acid profiles. This project aimed to decrease phlebotomy losses and painful procedures for our patients, eliminate unnecessary metabolic evaluations, and decrease healthcare expenditures for the hospital by decreasing the number of repeat blood spot cards sent. In addition, we aimed to reduce abnormal (false positive) NBS in the VLBW population from 66% to 43% within 12 months, given that 23% of our baseline results were abnormal due to an elevated amino acid profile alone.

## METHODS

### Setting

The UVa NICU, a part of an academic quaternary care center, conducted this study. The NICU admits 800 patients annually, of which approximately 100 are VLBW. The institutional review board approved this project as a QI initiative. The investigators obtained all data via the Epic (Epic Systems, Verona, Wis.) electronic health record.

### Data Collection

The investigators conducted an initial chart review to obtain the baseline abnormal NBS rate; this included reviewing all neonates admitted to our NICU between September 2018 and August 2019. The chart review aimed to gather information regarding gender, gestational age, birth weight, age (in hours) at the time of NBS collection, the number of NBSs collected on each patient, and the results of each NBS.

After implementation in July 2020, the investigators used the report function in Epic to identify all screens collected per month. All neonates’ NBS results were reviewed monthly until the end of our data collection through June 2021. Our target population for this intervention was VLBW neonates. However, we also included a separate analysis of all neonates admitted to the NICU to assess whether we could decrease our overall rate in addition to the rate of abnormal screens in VLBWs. Although our hypothesis postulates that TPN is causing an increase in abnormal NBS rates, the analysis presented includes all neonates in the NICU because the process of discussing the NBS on rounds was implemented for all patients. In addition, some patients intentionally had the NBS drawn at a different time before TPN started, which helped decrease our abnormal screen rate. After completing the data collection period, the investigators completed a chart review for all admissions to determine the rate of abnormal screens for all neonates on TPN at the time of NBS collection.

### QI Interventions

**Fig. 1.**
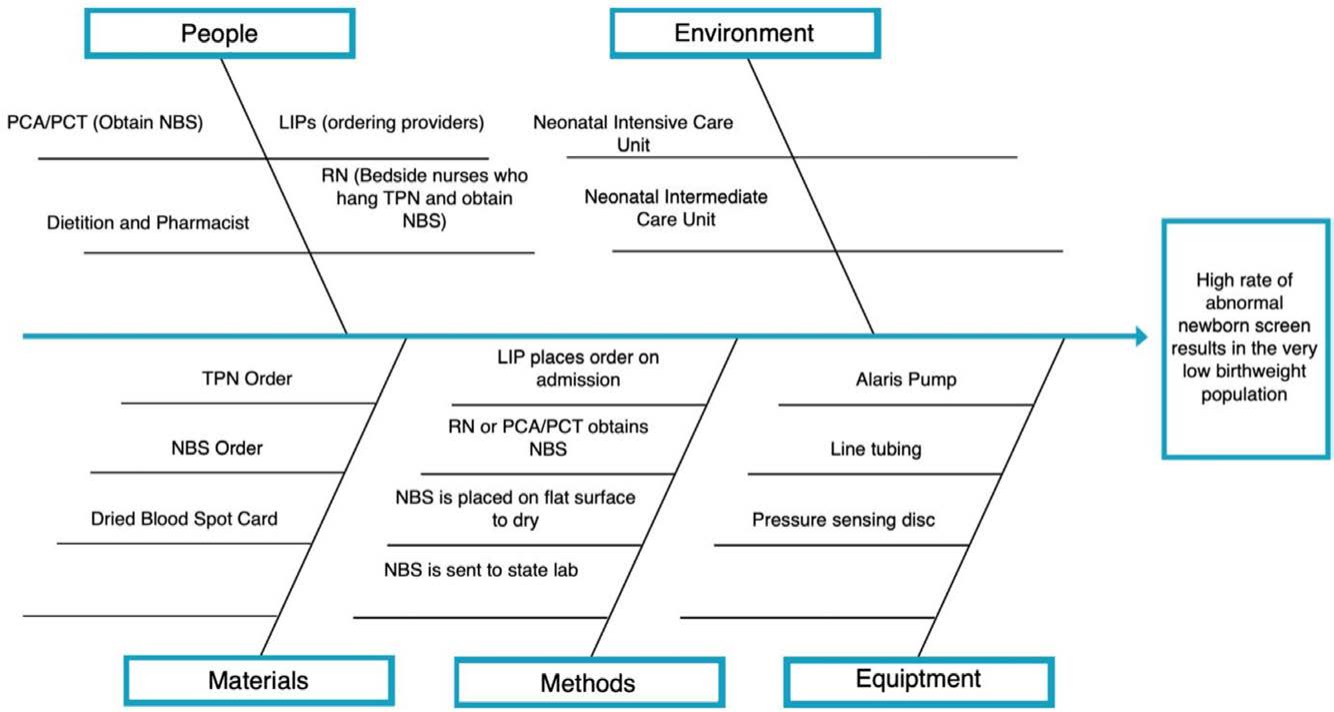
Cause and effect diagram (Fishbone diagram).

At the start of the project, the multidisciplinary team created a cause-and-effect (Fig. 1) to identify the key stakeholders, materials required, methods currently in place, and the equipment required. The stakeholders included a licensed independent practitioner who writes the order (MD, DO, NNP, or PA), the registered dietitian who ensures the TPN order meets adequate nutritional requirements, the pharmacist who verifies and fills the TPN order, the day and night shift nurses who manage the TPN, and the provider who draws the NBS (RN, or certified nursing assistant). A representative from these stakeholder groups helped form a multidisciplinary quality improvement (QI) team.

**Fig. 2.**
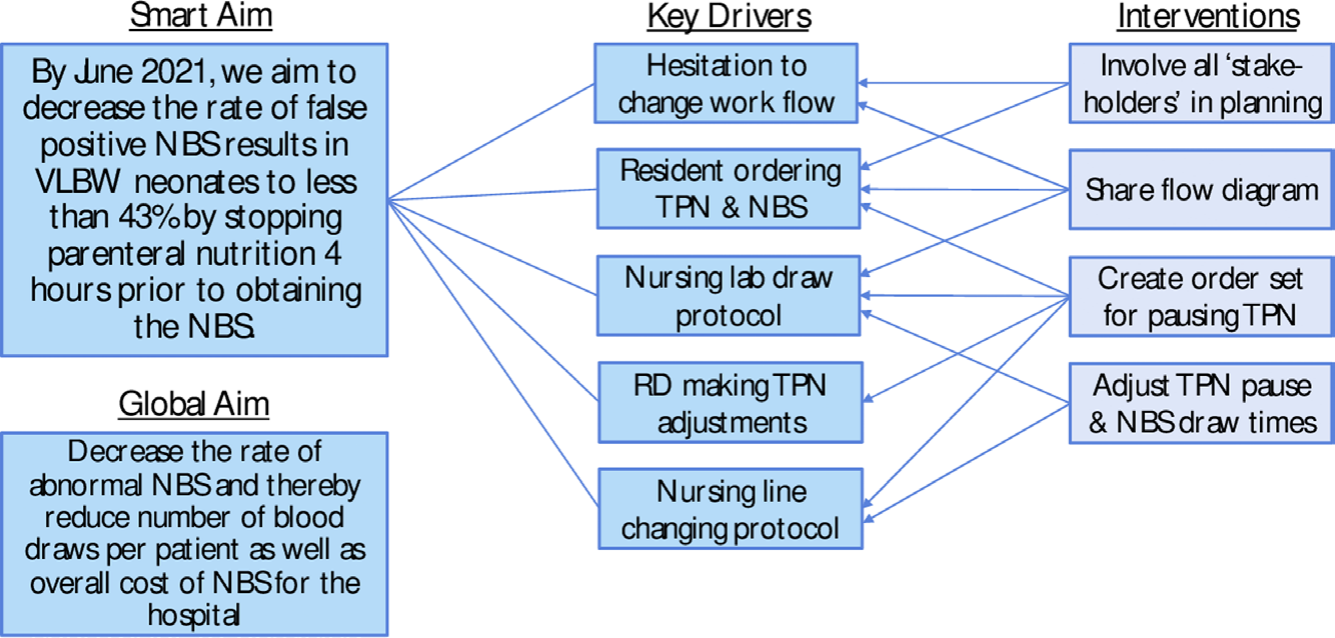
Key driver diagram.

This team then worked together and initially created a key-driver diagram (Fig. 2) to discuss how to implement change. Previous studies^4–6^ had shown that pausing TPN for 3 hours significantly reduced the abnormal NBS rate and hypothesized that pausing for a longer period could have additional benefits. Thus, the team paused TPN for 4 hours and ran dextrose-containing fluids to maintain euglycemia before collecting the NBS.

**Fig. 3.**
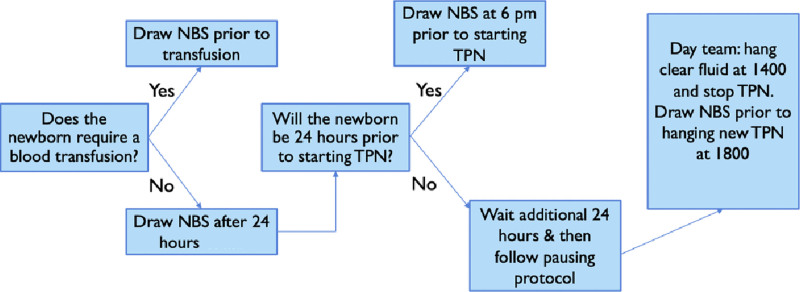
PDSA cycle 1 workflow.

To achieve ideal implementation, the team created and distributed a new NBS collection workflow (Fig. 3). The proposed workflow went through 3 main questions. First, does the neonate require a blood transfusion? Second, will the neonate be 24 hours old before starting TPN? Third, will the neonate be 24 hours of age before 1800 that day? With these 3 questions, the neonate could either get an NBS drawn before a transfusion, before starting TPN, or per the pausing protocol once they were > 24 hours of age.

The team also worked with Epic developers and created an Epic order set to help facilitate pausing the TPN and collecting the NBS at the end of the pause period (Appendix, Supplemental Digital Content, http://links.lww.com/PQ9/A406). The project’s balancing measure included incorrectly drawn NBS and nursing workflow satisfaction. The project’s process measure included the percentage of NBS drawn within 48 hours.

### PDSA Cycles

During PDSA (Plan, Do, Study, Act) cycle 1, nurses paused TPN at 1400 and collected the NBS at our standard infusion line change time of 1800. Implementation was a multistep process. First, education was given to attending, fellow, and resident physicians and the advance practice providers (neonatal nurse practitioners and physician assistants) on the new Epic order set and when to implement the TPN pause. Next, we communicated to the nursing staff, explaining the change via electronic mail and our weekly unit newsletter. More importantly, one representative from the group attended the morning (0910) and evening (2130) unit-based leadership huddles daily to remind everyone of the change and added the new workflow diagram to the huddle points.

**Fig. 4.**
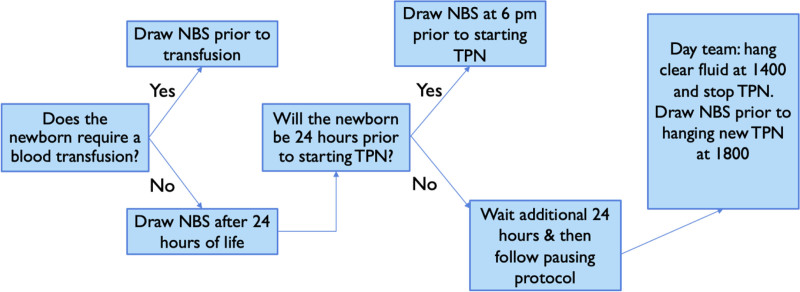
Improved workflow in PDSA cycle 2.

After 3 months, investigators surveyed the frontline staff for feedback. The survey identified a higher likelihood of error in the collection due to its proximity to shift change and other central line change responsibilities of the nurse. Additionally, drawing the NBS had typically been a night shift procedure, and day shift nurses were not as knowledgeable. This feedback led to PDSA cycle 2 with a revised workflow (Fig. 4). In the revised workflow, TPN was paused at 0000 to enable NBS collection at 0400 with routine morning laboratory work and helped divide the tasks between the day and night shift nurses and return the NBS collection to more experienced team members. At line change time at 1800, the dextrose-containing fluids are hung in line with the TPN, so when it is time to pause the TPN, the dextrose fluids can be started without breaking into the line. The investigators obtained cost data from the Virginia department of health as price per dried bloodspot card. They then estimated costs based on the number of cards sent as documented in Epic, which was limited to the fiscal year (July–June). Lipids were not paused during the intervention.

### Statistical Analysis

The percentage change in the rate of abnormal screens was analyzed using an unpaired *t* test and followed monthly using a P chart to assess for changes. The differences in the balancing measures were also analyzed using an unpaired *t* test to look for statistical significance.

## RESULTS

**Fig. 5.**
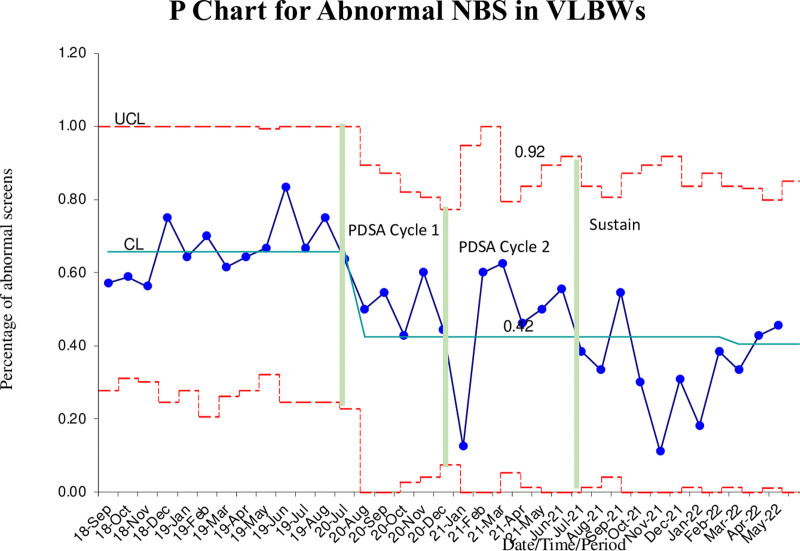
P chart for baseline and PDSA cycles.

Before initiation of the intervention, investigators obtained retrospective data on neonates admitted to the NICU (Neonatal Intensive Care Unit) at UVa from September 2018 to August 2019. During this time, there were 106 VLBW neonates admitted, with 6 dying before drawing the NBS and 8 had their NBS drawn elsewhere. Of the 92 remaining, 61 (66%) neonates had an abnormal NBS. None of the abnormal amino acid profiles were true positives; all patients had a normal repeat NBS or negative metabolic workup. During this baseline period, the NICU spent $21,114 on NBS blood spot cards in the VLBW population. Nearly, a quarter of the abnormal results were due to a neonate’s elevated amino acid profile while receiving TPN. No screens were incorrectly drawn, oversaturated, or insufficiently filled. However, the data did find that screens drawn after 48 hours occurred three and a half percent (3.5%) of the time. Pausing TPN for 4 hours before obtaining the NBS allowed us to decrease the rate of abnormal screens from 66% to 49% (*P* < 0.006; 95% CI, 0.04–0.27) (Fig. 5). The achievement of 10 out of 11 data points below the median and 6 in a row on our monthly P chart indicates a significant change from the intervention.

**Fig. 6.**
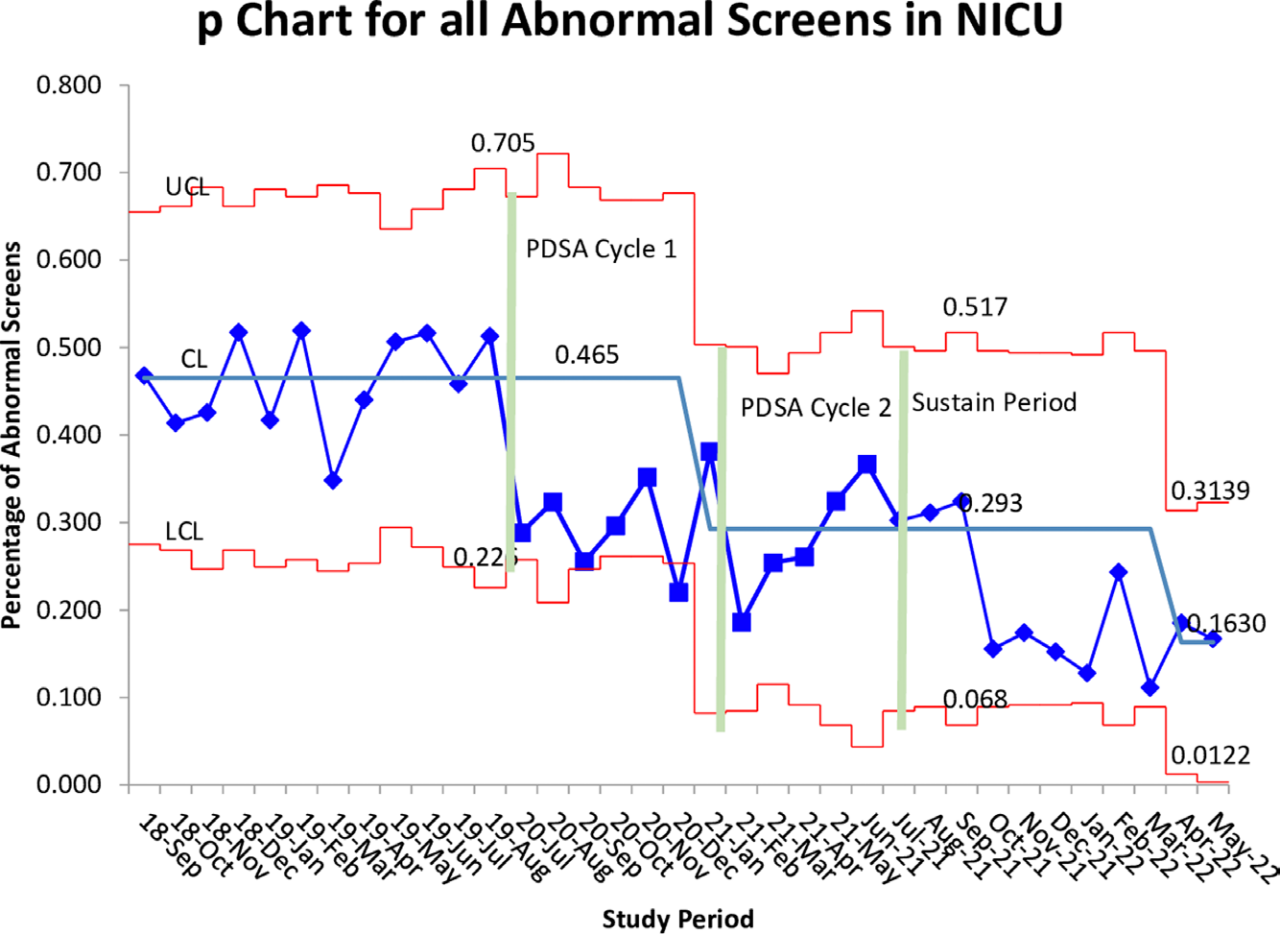
P chart data for baseline and PDSA cycles.

In addition to VLBWs, we looked at all NBSs drawn in the NICU during the study period. There were 645 NBSs drawn from September 2018 to August 2019. We found that the rate of overall abnormal NBS was 45.2%. After implementation, we decreased the abnormal rate to 28.8%; *P* < 0.0001; 95% CI, 0.06–0.51 (Fig. 6).

We also decreased hospital costs by decreasing the rate of abnormal screens. In 2018−2019 for 85 patients, the hospital spent $21,114 ($251.36 per patient) to perform the NBS in our VLBW population. In 2020−2021 for 84 patients, it cost $14,352 ($170.86 per patient). This change is a cost savings of $6762 in our VLBW population alone. The geneticist on call determined the decision to resend an NBS or to complete a metabolic workup.

Balancing and process measures followed included incorrectly drawn screens and screens drawn after 48 hours. Four percent (4%) of the NBSs were incorrectly drawn during the study period requiring repeat screens (*P* = 0.1925; 95% CI, –0.08 to 0.02). Seven percent (7%) of the screens were drawn after 48 hours (*P* = 0.3509; 95% CI, –0.10 to 0.04). We found no significant differences between our baseline data and our study period.

## DISCUSSION

In the 1990s, early administration of TPN became the standard of care for VLBW neonates.^7^ In our NICU, neonates weighing less than 2000 g, less than 35 weeks, or are term infants expected to be NPO for a minimum of 48 hours are started on TPN. Starter TPN can be ordered on admission for those neonates less than 1800 g, and all other neonates receive dextrose-containing fluid until the following day when they get custom TPN. This custom TPN can only be ordered before 1400 and arrives in the unit at 1800. The nursing staff hands the new TPN at 1800 before the end of the dayshift. These time constraints were the basis for our initial workflow.

The most challenging aspect of the project was creating a standard workflow in which all babies would have their NBS collected simultaneously. This challenge was difficult because neonates turn 24 hours of age at different times during the day, so we had to create a workflow that would prompt providers to order the TPN pause before the 24-hour mark. Initially, the team thought this would be easiest to remember if the nurse paused the TPN during dayshift after rounds and then restarted the TPN at 1800 when the nurse obtained a new bag of TPN from the pharmacy. This workflow was difficult for the nursing staff because it required them to complete extra tasks during an already busy time. This change led to TPN pausing errors (paused for less than 4 hours) and increased laboratory draws in those neonates not scheduled for a laboratory draw at 1800. The multidisciplinary team revised the protocol to pausing the TPN from 0000 to 0400 and obtaining the NBS with morning laboratories. This modification allowed the laboratory draw when the neonate was likely getting other blood tests and was not close to the change of shift. This change improved nursing satisfaction based on informal interviews and potentially decreased patient laboratory draws.

Although we have shown that this technique of pausing TPN has decreased our false positive rate of the NBS, one limitation of the study is that it only corrects the falsely elevated amino acid profile. As is seen in our control chart, there is a wide variability throughout the study. The researchers attribute this to the small number of VLBW admissions per month. In an emergent situation, such as before a blood transfusion, the NBS is drawn while the neonate receives parenteral nutrition, increasing the number of NBSs with a falsely abnormal amino acid profile. This leads to increased variability even if only 1 or 2 NBSs need to be collected with such a small denominator. In addition, it does not address the common thyroid hormone or congenital adrenal hyperplasia false positives, often leading to repeat NBS. Another limitation is that this method requires a TPN pause for 4 hours, and there is decreased protein delivery during this time. Furthermore, with the pause in TPN, nursing staff may forget to restart the TPN after pausing. This error has not been seen, but the risk exists.

Additionally, the providers prescribe the TPN for 24 hours, so the neonates miss 4 hours of nutrition. In the future, a trial of TPN for 20 hours could be attempted to help neonates get all the nutrition needed. Finally, a limitation of our study is that it increased the rate of incorrectly drawn NBSs. We have attributed this to a change in staff, and since investigators have trained new staff appropriately, the rate has decreased.

Since the end of the study period and throughout the sustain period, we have seen a decrease in the percentage of abnormal NBSs. A new change in the centerline occurred at the end of the sustain period. The researchers feel this is due to increased familiarity with the protocol, and nursing re-education completed in July 2021. A reminder about the protocol went out in the weekly email sent to staff, and the protocol was discussed at the huddle twice a day with the staff. These calls to action likely led to the continued improvement.

In conclusion, we decreased the rate of abnormal NBSs in all neonates in addition to the previously studied VLBW population using QI methodology. The researchers were able to create and sustain a new workflow and implement change for all patients, not just VLBW neonates. This work will be helpful for other NICUs looking to implement a new screening process and shows that the process of pausing TPN before drawing the NBS has the potential to benefit all types of patients on TPN, not just those who are VLBW. In our tertiary care NICU, we plan to improve our workflow and efficiency, as evidenced by our sustain-period to continue our positive results while decreasing our incorrectly drawn screens and screens drawn after 48 hours.

## ACKNOWLEDGMENTS

Assistance with the study: Dr. Jonathan Swanson assisted with statistical analysis

## DISCLOSURE

The authors have no financial interest to declare in relation to the content of this article.

## Supplementary Material


