# Supplementary material for: Pausing TPN to Decrease Abnormal Newborn Screens: A NICU Quality Initiative
Source: Pediatr Qual Saf. 2022 Sep 15;7(5):e595. doi: 10.1097/pq9.0000000000000595 (PMC9478258; doi:10.1097/pq9.0000000000000595)
Supplement: Supplementary file 1 [file pqs-7-e595-s001.pdf]

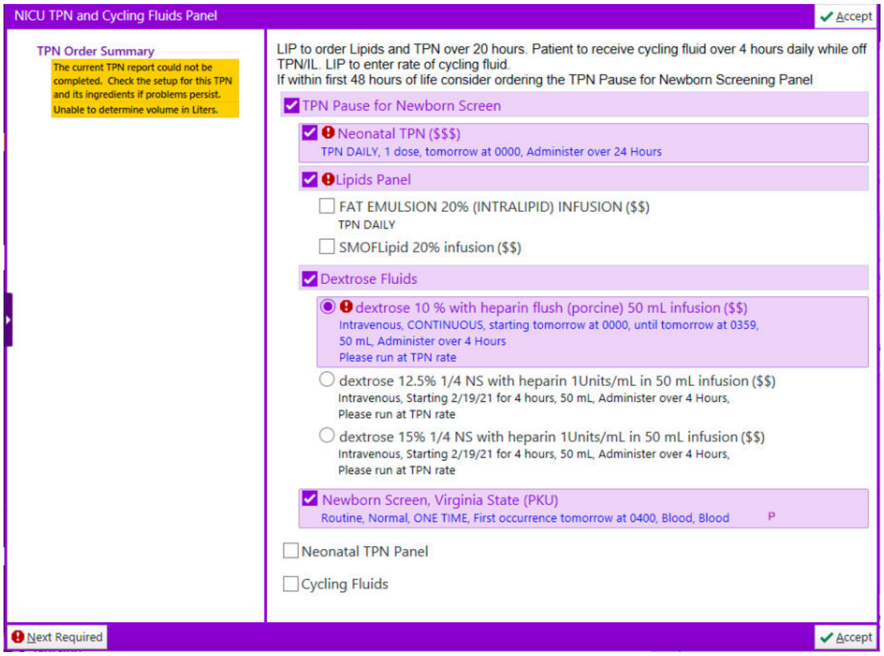

Appendix A: Revised Epic order set
